# CD4^+^ and CD8^+^ T cells are not the main driver of Lassa fever pathogenesis in macaques

**DOI:** 10.1172/jci.insight.199235

**Published:** 2025-09-25

**Authors:** Jérémie Prévost, Nikesh Tailor, Geoff Soule, Jonathan Audet, Yvon Deschambault, Robert Vendramelli, Jessica Prado-Smith, Kevin Tierney, Kimberly Azaransky, Darwyn Kobasa, Chad S. Clancy, Heinz Feldmann, Kyle Rosenke, David Safronetz

**Affiliations:** 1 Special Pathogens, National Microbiology Laboratory, Public Health Agency of Canada, Winnipeg, Manitoba Canada.; 2 Rocky Mountain Veterinary Branch, National Institute of Allergy and Infectious Diseases, NIH, Hamilton, Montana, USA.; 3 Department of Medical Microbiology and Infectious Diseases, University of Manitoba, Winnipeg Manitoba, Canada.; 4 Laboratory of Virology, National Institute of Allergy and Infectious Diseases, NIH, Hamilton, Montana, USA.

**Keywords:** Microbiology, Public Health, Virology, T cells

## Abstract

Empirical data from survivors of Lassa fever and experimental disease modeling efforts, particularly those using mouse models, are at odds with respect to T cell–mediated pathogenesis. In mice, T cells have been shown to be imperative in disease progression and lethality, whereas in humans, an early and robust T cell response has been associated with survival. Here, we assessed the role of CD4^+^ and CD8^+^ T cells on disease progression and severity of Lassa virus infection in a nonhuman primate model. Using an antibody-mediated T cell depletion strategy prior to and after inoculation, we were able to examine Lassa virus infection in the absence of specific T cell responses. In animals depleted for either CD4^+^ or CD8^+^ T cells, Lassa virus infection remained uniformly lethal, with only a slight delay in disease progression was observed in the CD4-depleted group when compared with nondepleted controls. Milder pulmonary pathology was noticed in the absence of CD4^+^ or CD8^+^ T cells. Overall, our findings suggest that T cells have a limited effect on the development of Lassa fever in nonhuman primates.

## Abstract 2

Using antibody-based depletions we demonstrate that deleterious T cell responses are not a primary driver in Lassa virus severity in a non-human primate model

## Introduction

Lassa virus (LASV, genus *Mammarenavirus*, family *Arenaviridae*) is a zoonotic virus that is endemic in several countries in Western Africa, including Sierra Leone, Liberia, Guinea, and Nigeria as well as parts of Mali, Ghana, Cote d’Ivoire, Togo, and Benin (1, 2). The primary reservoir for LASV is the commensal multimammate rat (*Mastomys natalensis*) (3). Although human-to-human transmission of LASV is well documented, the majority of human infections likely come directly from infected rodents (4).

Unlike most etiological agents of classical viral hemorrhagic fever, LASV infection can manifest in a variety of clinical presentations (5, 6). The majority of LASV infections are asymptomatic or mild in nature; however, in approximately 20% of cases, infection leads to severe disease associated with hemorrhagic manifestations and multiorgan failure, a condition referred to as Lassa fever (LF) (7, 8). Severe cases of LF are characterized by vomiting, diarrhea, increased levels of liver enzymes (ALT and AST), and elevated hematocrit (9, 10). Abdominal and retrosternal pain, edema of the face and neck, enlarged lymph nodes, and/or hemorrhage in the conjunctiva or mucosal surfaces are particularly indicative of a poor prognosis (11). In fatal cases, fever is maintained and rapid deterioration occurs over the first 2 weeks and is associated with hypovolemia, hypotension, pleural effusion, ascites, anuria, bleeding (gums, nose, intestine, or vagina), and platelet dysfunction. Acute neurological changes are also common and can include signs of encephalopathy such as generalized seizures, dystonia, and neuropsychiatric changes.

Estimates vary, though upward of 300,000 people may become infected with LASV on an annual basis, making LASV one of the most prominent etiological agents of hemorrhagic fever worldwide (6, 12). In addition to annual outbreaks in West Africa, exported cases of LF have been reported in North America, Europe, and Asia, making LASV a global health concern (13).

Although the fatality rate is estimated at < 5% of all suspected infections, it increases dramatically in nosocomial settings, and outbreaks where mortality rates exceeding 50% have been documented (14). Recent statistics from Nigeria, which has been experiencing increasingly severe and prolonged outbreaks of LF since 2015, show case fatality rates exceeding 20% in confirmed or suspected cases (9). Currently, there are no approved therapeutics to treat LF, although ribavirin has been used as an off-label treatment option with mitigated results (15). There are also no approved vaccines to prevent LASV infection, though some promising vaccine candidates are in advanced clinical trials (16, 17).

The development and thorough characterization of animal models of disease is a critical step in elucidating pathogenesis of disease and the preclinical evaluation of medical countermeasures against high-consequence pathogens like LASV. To this end, several immunocompetent animal models have been described for studying LASV pathogenesis with guinea pigs (inbred strain 2 or 13, or outbred Hartley animals) and nonhuman primates (NHPs) being the most commonly used (18, 19). While useful as a screening model, the disease manifestations in guinea pigs do not faithfully recapitulate those seen in patients; therefore, the preferred model for pathogenesis as well as vaccine and therapeutic studies are NHPs — most commonly cynomolgus macaques (18, 20). Similar to humans, severe disease manifestations in NHPs can differ. Although the majority of studies conducted in NHPs to date have focused on infection with LASV strain Josiah (clade IV, originating from Sierra Leone), recent studies with isolates from Mali, Liberia, Nigeria, and Togo have all highlighted differing disease progressions, clinical manifestations and hematological/biochemical abnormalities (21–23). Despite a wealth of studies in both animal models, the underlying mechanisms of LF-like disease in these models remain to be elucidated.

Immune-mediated pathogenesis has been implicated in disease severity associated with many etiological agents of VHF, including LASV, though the immunological basis is not well understood. Survivors of LF often exhibit robust LASV-specific T cell responses, and this has led to the belief that T cell–mediated immunity is critical for protection (24–26). Alternatively, in murine models of LF, the prevailing hypothesis is that CD8^+^ T cell responses contribute to disease progression and immune-mediated pathology, while CD4^+^ responses are less involved (27–29). In the current study, we sought to assess the role of circulating T cells in the gold-standard NHP model. Using an established T cell–specific antibody-based depletion protocol (30), we eliminated CD4^+^ or CD8^+^ T cells from groups of 4 NHPs prior to and after LASV challenge. Disease progression was assessed and compared with control, nondepleted animals daily — before and after challenge — with hematological, biochemical, and virology parameters measured at regular intervals. Although there was a slight delay in onset and severity after LASV infection in the CD4^+^-depleted group, overall our findings suggest that circulating CD4^+^ and CD8^+^ T cells do not have a direct effect on the development of LF in the NHP model.

## Results

### In vivo circulating T cell depletion efficiency.

**Figure 1:**
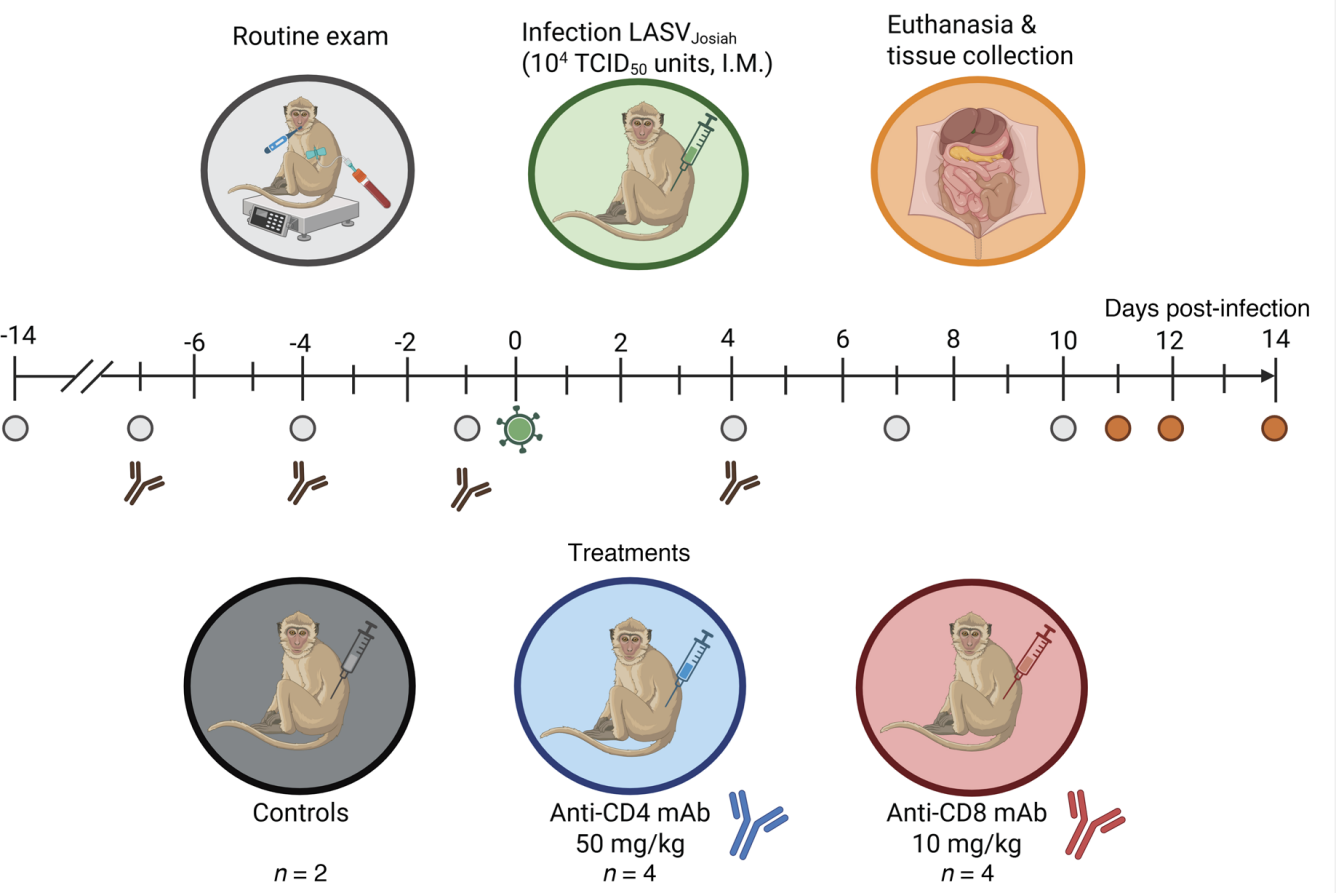
Experimental layout for T cell depletion in LASV-infected cynomolgus macaques. Male cynomolgus macaques (*Macaca fascicularis*) were administered with CD4- or CD8-depleting monoclonal antibodies (mAb) at day 7, 4, and 1 before infection and at day 4 after infection (*n* = 4 per group). Control animals were mock-treated with saline (*n* = 2). All NHPs were infected with 1 × 10^4^ TCID_50_ units of Lassa virus (LASV) strain Josiah (clade IV) by intramuscular (i.m.) injection. Animals were monitored twice daily, routine exams (vital signs and blood collection) were performed at multiple time points before and after infection (–14, –7, –4, –1, 4, 7, and 10 DPI) and necropsy exams were performed on terminally ill animals (11–14 DPI). Image created using Biorender.com.

**Figure 2:**
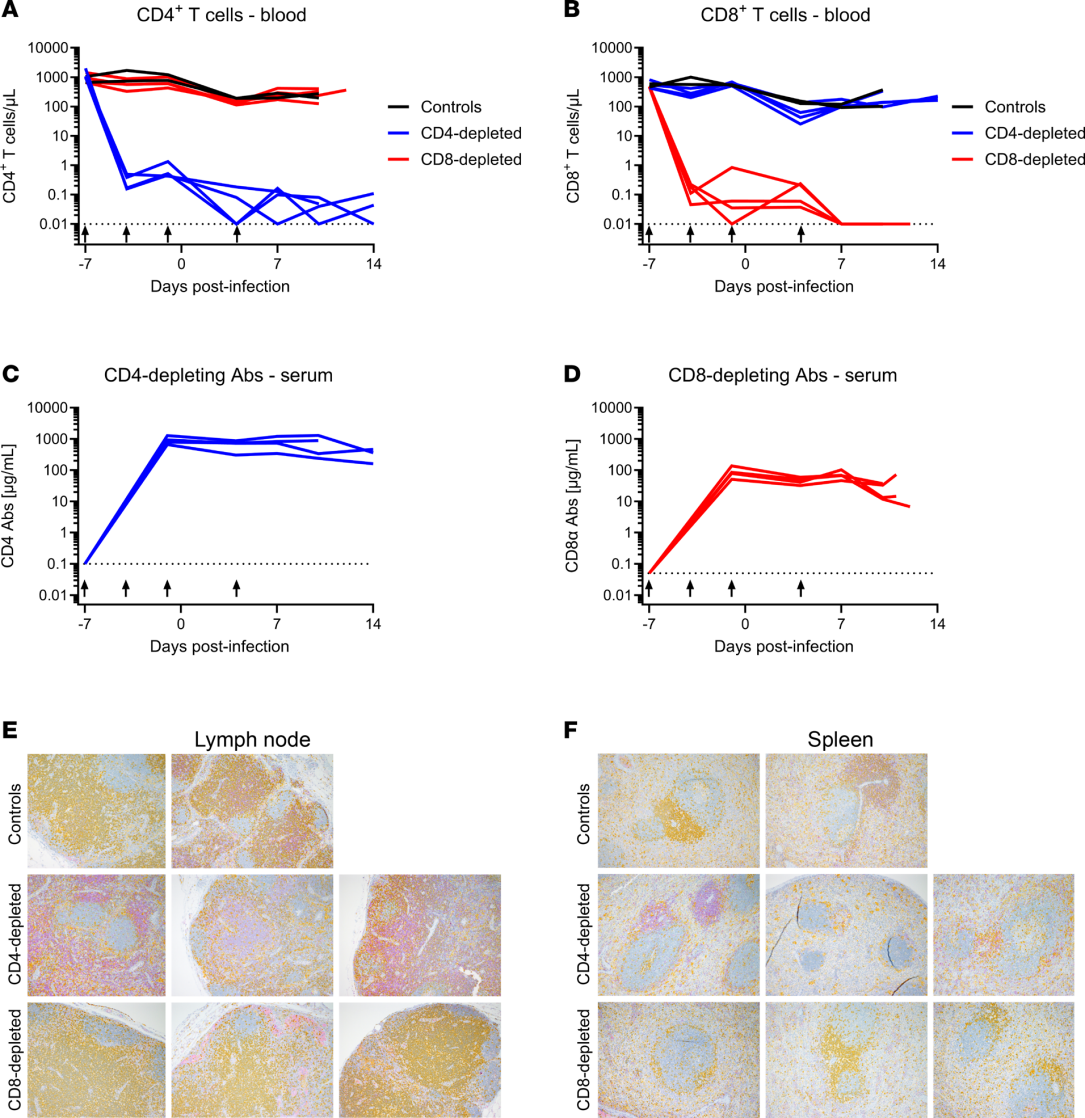
T cell depletion efficiency in cynomolgus macaques. (**A** and **B**) Absolute counts of circulating CD4^+^ (**A**) and CD8^+^ (**B**) T cells were monitored from fresh EDTA-treated whole blood during routine exams (–7, –4, –1, 4, 7, and 10 DPI) and terminal necropsy exams using flow cytometry. (**C** and **D**) Depleting antibodies (Abs) targeting CD4 (**C**) or CD8α (**D**) were monitored from serum at the same timepoints using an in-house ELISA. (**A**–**D**) Arrows indicate the days when depleting antibodies were administered. Data are represented as connecting lines for each individual animal. (**E** and **F**) At the time of necropsy, lymphoid tissues including lymph node (**E**) and spleen (**F**) were collected for assessment of T cell depletion in tissues. IHC staining of CD4^+^ cells (yellow) and CD8^+^ cells (purple) are shown from representative sections from control (*n* = 2), CD4-depleted (*n* = 3), and CD8-depleted (*n* = 3) animals. Total original magnification, ×100.

Groups of 4 animals were depleted for CD4^+^ or CD8^+^ T cells prior to LASV challenge via antibody treatments using a previously established specific depletion strategy (Figure 1) (30). To ensure the depletions were sustained throughout the acute phase of infection, animals were similarly depleted once after challenge. A control group of 2 animals was included in the experiment that did not receive any depleting antibodies, but it was examined and sampled simultaneously under the same conditions. Depletion efficiencies were regularly monitored by flow cytometry on fresh whole blood collected on exam days (Figure 2, A and B). Following administration of the anti-CD4 antibody, the population of circulating CD4^+^ T cells dropped from 1283 cells/μL to 0.3 cells/μL within 3 days of the initial treatment (Figure 2A and Supplemental Figure 2A; supplemental material available online with this article; https://doi.org/10.1172/jci.insight.199235DS1). At the time of viral challenge, CD4^+^ T cells were abrogated, with no rebound detected throughout the course of the experiment. As expected, the CD8^+^ T cell population in the four CD4^+^-depleted animals remained relatively constant. Similarly, the 4 NHPs specifically treated with CD8-depleting antibodies showed a marked decrease in CD8^+^ T cell populations immediately after the first antibody treatment (Figure 2B and Supplemental Figure 2A). In these animals, CD8^+^ T cell populations dropped from 469 cells/μL to 0.1 cells/μL 3 days later. At the time of LASV challenge, CD8^+^ T cells were almost undetectable and remained so throughout the course of the study. The CD4^+^ T cell population in the NHPs specifically depleted for CD8^+^ T cells remained relatively unchanged. At between 3 and 5 log_10_ reductions in counts, the specific T cell depletions were highly significant with *P* values lower than 1 × 10^–16^. Of note, the depletion of other CD8^+^ cell populations, such as NK and NKT cells, was observed specifically in CD8-depleted animals (Supplemental Figure 2, B–E).

Depleting antibody levels in serum samples remained stable throughout the course of the infection, with concentrations around 1,000 μg/mL for the CD4-depleting mAb and 100 μg/mL for the CD8-depleting mAb (Figure 2, C and D). The effect of the specific T cell depletions extended beyond circulating CD4^+^ and CD8^+^ cells and into the lymphoid tissues (Figure 2, E and F). CD4 depletion resulted in skewing of the total lymphocyte population toward CD8^+^ lymphocytes, with a relative depletion of CD4^+^ lymphocytes in the lymph nodes and spleen. In both control and CD8-depleted groups, the lymphocyte population was skewed toward CD4^+^ lymphocytes with low to rare CD8^+^ lymphocytes (which could include NK, NKT, and CD8^+^ T cells) noted in evaluated sections.

### Disease progression and outcome in T cell–depleted NHPs.

**Figure 3:**
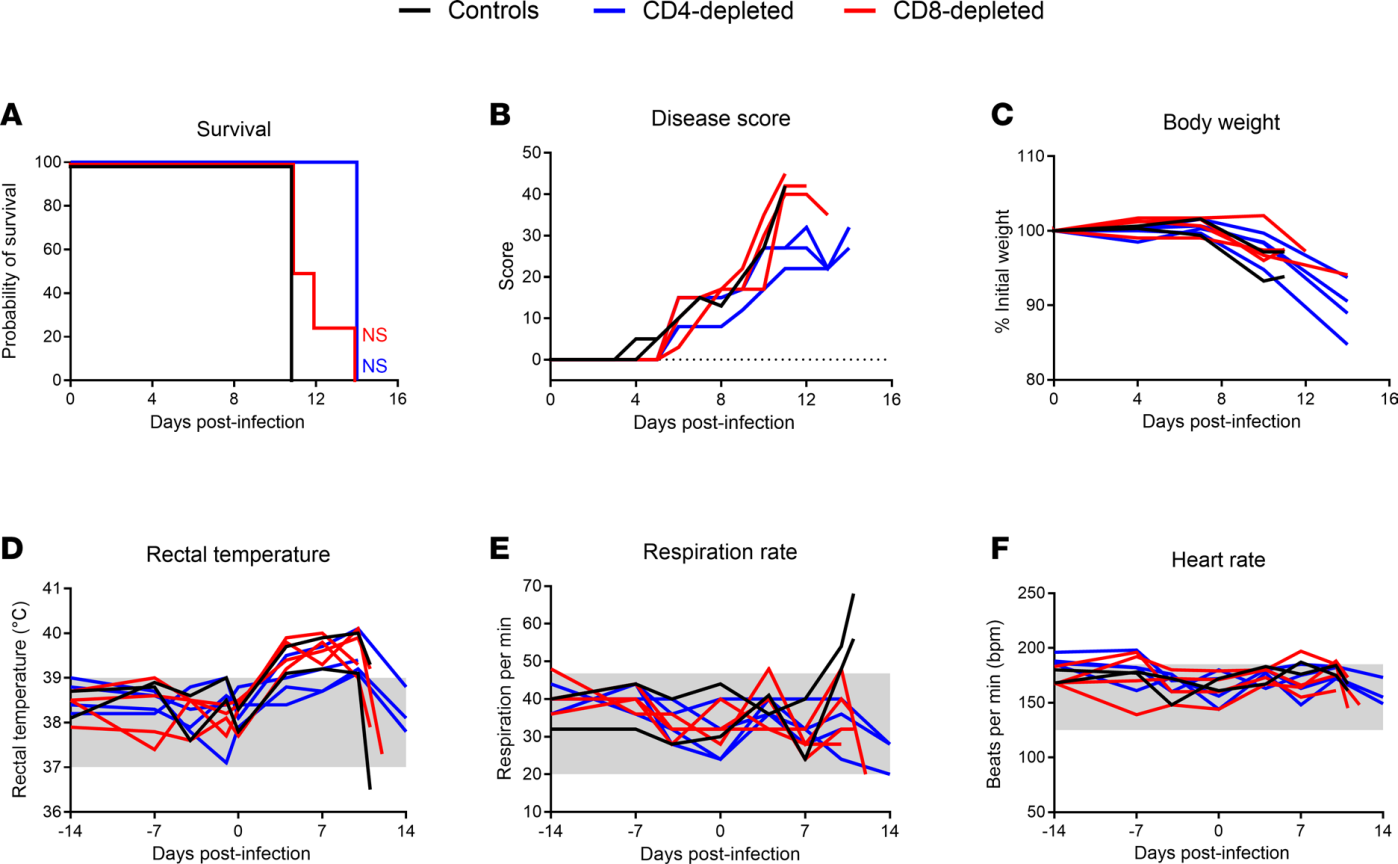
Disease progression of Lassa fever in T cell–depleted cynomolgus macaques. (**A**–**F**) CD4-depleted, CD8-depleted, and control groups of cynomolgus macaques were infected intramuscularly with 1 × 10^4^ TCID_50_ units of LASV Josiah (clade IV) and measured for signs of disease survival (**A**), disease clinical score (**B**), and weight loss (**C**), as well as vital signs including rectal temperature (**D**), respiration rate (**E**), and heart rate (**F**). (**A**) Statistical significance was calculated a log-rank test with a Holm-Sidak post hoc test. (**B**–**F**) Data are represented as connecting lines for each individual animal. Where available, normal ranges are indicated by gray shading area (55).

Prior to challenge, during the antibody-mediated depletions, all animals appeared normal with no signs of adverse reactions to the treatment (Figure 3). The first sign of infection in most animals was elevated temperatures, which occurred by 4 days postinfection (DPI), approximately 2 days before physical signs of disease were apparent. Elevated temperatures persisted in these animals until the terminal stage of disease, at which point a precipitous drop was noted in many of the animals (Figure 3D). The exceptions to this were 2 animals in the CD4-depletion group—which experienced slight elevations in body temperature—that did not exceed the normal range for this species (≤39°C). Beginning around day 6 after challenge, depleted and nondepleted control animals all begin to physically manifest signs of infection (Figure 3B). Initially, these signs included hunched posture, piloerection, and reduced activity, which progressed to include inactivity, depression, and reluctance to move as well as anorexia by day 10. At the terminal stages of disease, facial swelling and ataxia was apparent in most animals. Although disease progression in the CD4-depleted animals appeared slower after day 10 after challenge, indicators of severe disease were apparent in all 4 animals, resulting in the decision to euthanize these animals. Coinciding with inappetence, weight loss was noted in many animals, particularly those in the CD4 depletion group (Figure 3C). Heart rate appeared consistent throughout the course of the study across the 3 groups (Figure 3F). The perimortem respiration rate was elevated in the control, nondepleted animals, which was consistent with the presence of pleural effusion, petechial hemorrhage, and pulmonary pathological abnormities noted below (Figure 3E and Supplemental Figure 3). Overall, the predetermined humane endpoint in the nondepleted control animals was reached on day 11 after challenge, whereas in the CD8-depleted animals, mean time to euthanasia was day 12 (range 11–14 DPI). All four CD4-depleted animals had a delayed time to lethal disease, which occurred at day 14 for the entire group (Figure 3A). Pairwise comparisons of time to lethal disease found no significant difference between the control nondepleted group and the CD4- or CD8-depleted animals (*P* = 0.07 and *P* = 0.26, respectively). A meta-analysis for the survival rates of historical controls (cynomolgus macaques infected with LASV Josiah; *n* = 43) from 10 previous studies show that the median time to death is 12 DPI with an interquartile range of 11–14 DPI (21, 22, 31–38). Statistical analyses reveal no significant difference between historical controls compared with CD4-depleted, CD8-depleted, or control animals from the current study (Supplemental Figure 4).

### Hematology, coagulation, and serum biochemistry.

**Figure 4:**
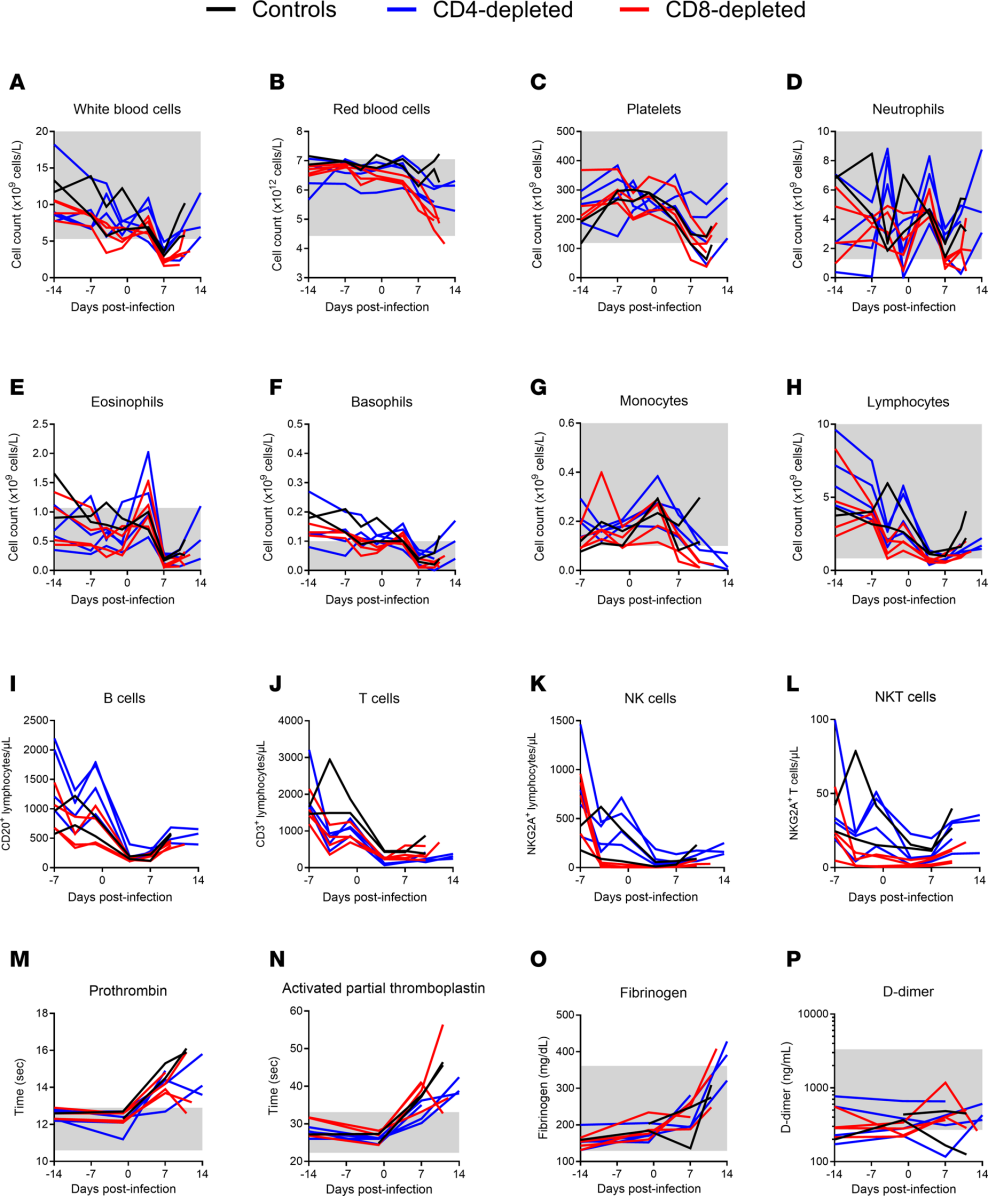
Hematological and coagulation parameters of T cell–depleted LASV-infected cynomolgus macaques. (**A**–**L**) EDTA-treated blood samples collected from NHPs at regular intervals before and after infection were monitored for absolute counts of white blood cells (**A**), red blood cells (**B**), platelets (**C**), neutrophils (**D**), eosinophils (**E**), basophils (**F**), monocytes (**G**), and lymphocytes (**H**) using a HM5 hematology analyzer. (**I**–**L**) Lymphocyte populations (B cells, T cells, NK cells, NKT cells) were monitored by flow cytometry. (**M**–**P**) Citrate plasma obtained from NHP blood samples were monitored for prothrombin (**M**), activated partial thromboplastin (**N**), and fibrinogen (**O**) using the Satellite Max analyzer, as well as D-dimer by ELISA (**P**). Data are represented as connecting lines for each individual animal. Where available, normal ranges are indicated by gray shading area (56).

**Figure 5:**
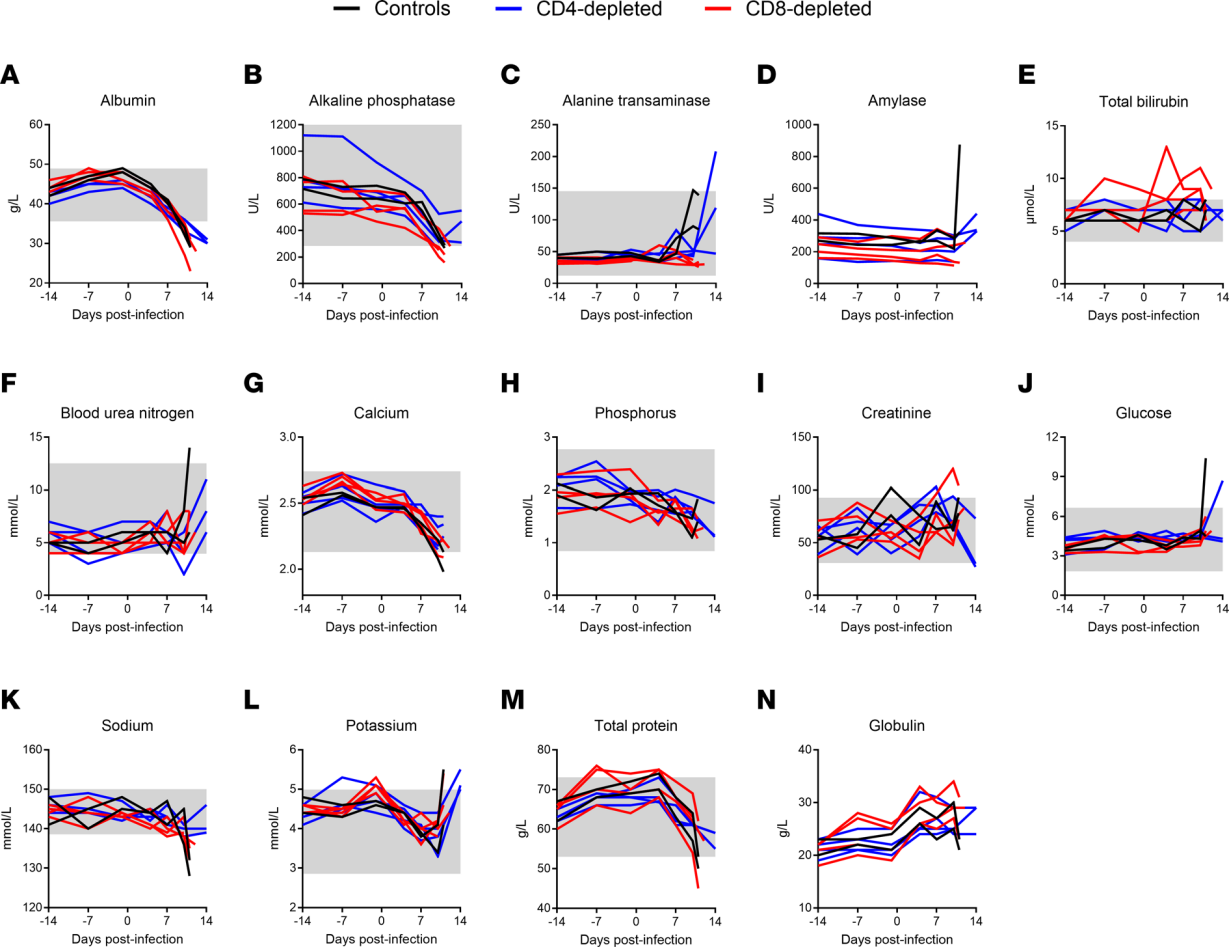
Blood biochemical parameters of T cell–depleted LASV-infected cynomolgus macaques. (**A**–**N**) Serum samples collected from NHPs at regular intervals before and after infection were monitored for levels of albumin (**A**), alkaline phosphatase (**B**), alanine aminotransferase (**C**), amylase (**D**), total bilirubin (**E**), blood urea nitrogen (**F**), calcium (**G**), phosphorus (**H**), creatinine (**I**), glucose (**J**), sodium (**K**), potassium (**L**), total protein (**M**), and globulin (**N**) using a VS2 biochemistry analyzer. Data are represented as connecting lines for each individual animal. Where available, normal ranges are indicated by gray shading (56).

The overall trends in the hematological, coagulation and biochemical parameters monitored in the study did not differ across the CD4- or CD8-depletion groups and the nondepleted control animals, and after challenge, they were essentially as previously described for LASV infection in NHPs (21, 22) (Figures 4 and 5). The most notable hematological change was a sharp decrease in platelets beginning as early as 4 DPI and approaching levels consistent with thrombocytopenia (<150 × 10^9^/L) between 7 DPI and terminal disease (Figure 4C). A transient increase of innate immune cells (neutrophils, eosinophils, basophils, and monocytes) was observed at 4 DPI, returning to normal levels thereafter (Figure 4, D–G). Lymphopenia (<1 × 10^9^/L) was also observed early after challenge and until terminal disease, showing decreasing counts essentially in all subpopulations monitored (Figures 4, A and H–L). RBC counts were largely unchanged in the control animals; however, decreases in RBC were observed in both depletion groups, with more distinct drops observed in the CD8^+^ depleted animals (Figure 4B).

Consistent with the hematological observations, particularly the decreasing platelet counts observed, coagulation parameters were indicative of coagulation disorders in all 3 groups. A modest increase in prothrombin time (PT) (2–4 seconds) overlapped by more substantial increases in activated partial thromboplastin time (aPTT) were observed following LASV challenge and most notable after 7 DPI (Figure 4, M and N). Fibrinogen concentrations also increased along the same timeline, though increases in fibrinogen degradation products, as measured by D-dimers, were not observed (Figure 4, O and P).

Biochemically, common features of LASV infection were similar in the 3 groups in the current study (Figure 5). Specifically, decreasing serum concentrations of total protein (TP) as well as hypoalbuminemia (<35 g/L), hypocalcemia (<2.2 mmol/L), and hyponatremia (<135 mmol/L) were common in depleted and nondepleted NHPs. Other similarities across the groups include decreased alkaline phosphatase as well as sharp increases of blood urea nitrogen (>7.5 mmol/L) and potassium (>5 mmol/L) immediately perimortem. Total bilirubin (TBIL) was relatively unchanged in the CD4-depleted and control animals, though it was elevated (>8 μmol/L) after the challenge in the CD8-depleted animals. Conversely, alanine transaminase (ALT) was elevated (>100 U/L) in both the CD4-depleted and control animals after challenge, but it remained relatively unchanged in the CD8-depleted NHPs. Together, this suggests that both groups of T cell–depleted animals also show signs of liver disease, a hallmark of LF.

### Host immune responses.

**Figure 6:**
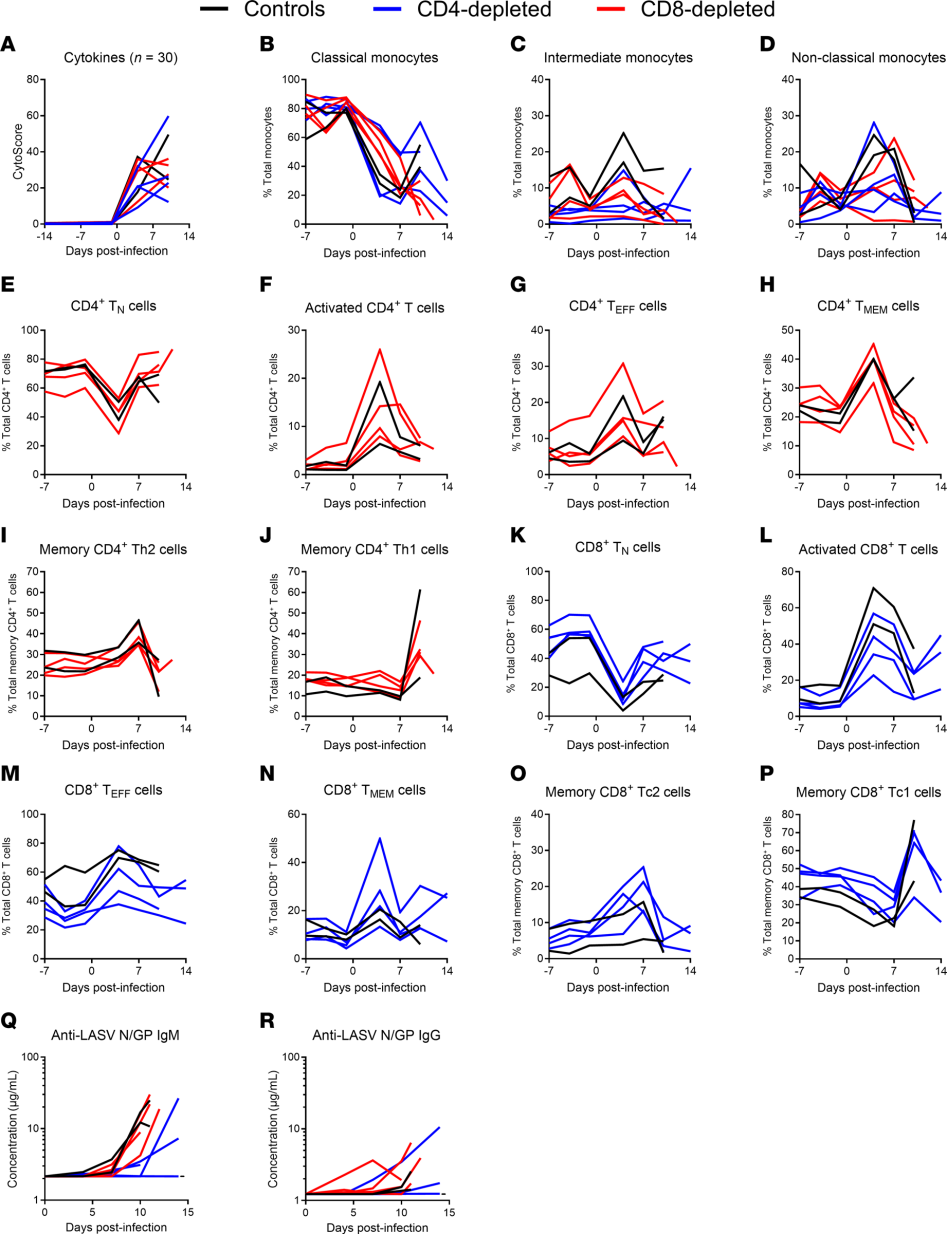
Host immune responses of T cell–depleted LASV-infected cynomolgus macaques. (**A**) Serum samples collected from LASV-infected NHPs at regular intervals before and after infection were monitored for the presence of 30 cytokines/chemokines using a multiplex fluorescent bead-based immunoassay. Cytokine scores (CytoScore) were calculated as the linear combination of all 30 analytes. (**B**–**P**) Percentages of circulating monocyte (**B**–**D**), CD4^+^ T cell (**E**–**J**), and CD8^+^ T cell (**K**–**P**) populations were monitored from fresh EDTA-treated whole blood during routine exams (–7, –4, –1, 4, 7, and 10 DPI) and terminal necropsy exams using flow cytometry. (**B**–**D**) The different monocyte populations were characterized by a differential expression of CD14 and CD16. (**E**–**P**) The different T cell populations were characterized by a differential expression of CD45RA and CCR7 (T_N_, T_EFF_, T_MEM_) and CD69 (activated) as well as CXCR3 and CCR4 (Th1/Tc1, Th2/Tc2). Tc, cytotoxic T cells; T_EFF_, effector T cells; T_MEM_, memory T cells; T_N_, naive T cells. (**Q** and **R**) Serum samples collected from LASV-infected NHPs at regular intervals after the infection were monitored IgM (**Q**) and IgG (**R**) antibody responses mediated against LASV nucleoprotein (N) and glycoproteins (GP) using a commercial ELISA assay. Dashed lines represent the limit of detection of the assay. (**A**–**R**) Data are represented as connecting lines for each individual animal.

Serum immune mediators including cytokines and chemokines were monitored in serial samples collected during scheduled exams using a 30-plex fluorescent bead-based immunoassay (Figure 6A and Supplemental Figure 5). To evaluate the magnitude of the cytokine storm using a single parameter, we calculated an integrated cytokine score from the panel of 30 analytes. The overall cytokine level increased drastically at 4 DPI and was still elevated thereafter (Figure 6A). Similar activation patterns for most proinflammatory cytokines (including G-CSF, GM-CSF, IFN-α, IFN-γ, IL-1β, IL-2, IL-5, IL-7, IL-12p70, IL-17A, IL-23, sCD40L, TNF-α), antiinflammatory cytokines (including IL-1RA, IL-4, IL-10, and IL-13), and immune-modulating chemokines (including CCL2, CCL3, CCL4, CCL11, CXCL8, CXCL9, CXCL10, CXCL11, CXCL12, and CXCL13) monitored were observed across the 2 depletion and the nondepletion control groups (Supplemental Figure 5). The majority of analytes remained unchanged during the depletion phase of the experiment and peaked between 4 and 10 DPI. Overall, the induction of a cytokine storm was observed across all 3 groups.

In-depth characterization of the dynamics of immune cell populations was performed on fresh blood samples throughout the experiment using multiparametric flow cytometry (Figure 6, B–P). Importantly, major changes were observed after infection in the monocytic and lymphocytic populations, without clear difference between depleted and nondepleted groups. Before the infection, classical monocytes were the dominant population (around 80% of total monocytes), but they were rapidly replaced in circulation by CD16^+^ monocytes (intermediate and nonclassical) within 4 DPI (Figure 6, B–D). Similarly, naive CD4^+^ and CD8^+^ T cells (T_N_), which accounted for the majority of circulating T cells before infection, were also rapidly replaced by activated T cells at 4 DPI (Figure 6, E, F, K, and L). These activated T cells mainly displayed effector or memory phenotypes, with an increase in Th2/Tc2 polarization at 7 DPI, followed by a switch to Th1/Tc1 polarization at 10 DPI (Figure 6, G–J and M–P). In the end, CD4 depletion did not seem to affect CD8^+^ cellular responses and vice versa for the effect of CD8 depletion on CD4^+^ cellular responses.

Humoral responses against the combined LASV nucleoprotein and prefusion glycoproteins were assessed on serial serum samples using anti-LASV IgM and IgG ELISAs (Figure 6, Q and R). Modest increases in IgM specific for the combination antigens were noted by 7 DPI in the control and CD8-depleted animals. Two of the CD4^+^-depleted animals also exhibited slight increases in IgM concentrations, though only after 10 DPI and in samples collected perimortem. In comparison, levels of IgG against the same antigens remained relatively low to undetectable in these animals, and at no point were neutralizing antibodies against LASV GP detected.

### Viral burden.

**Figure 7:**
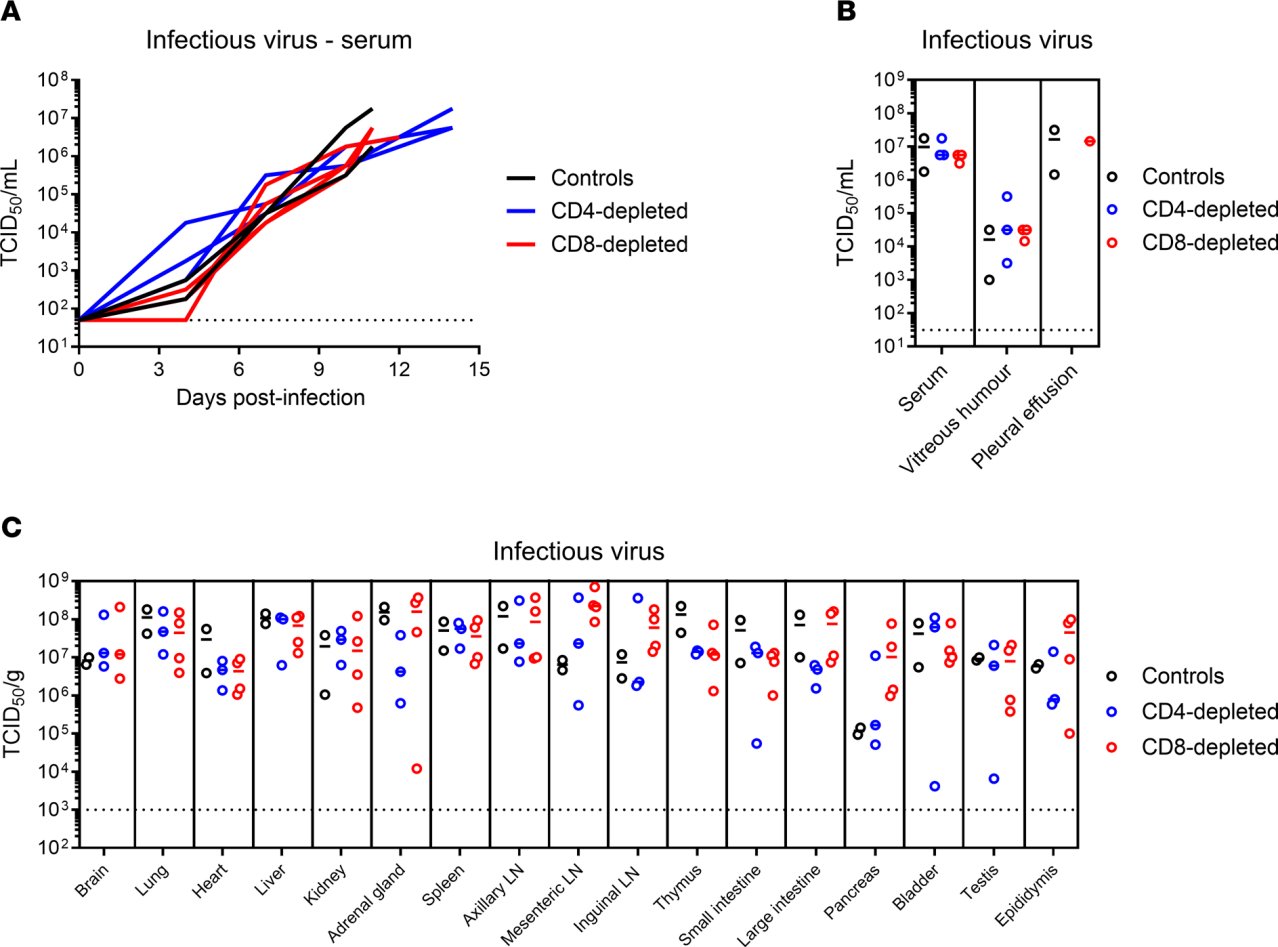
Viral burden in T cell–depleted LASV-infected cynomolgus macaques. (**A**) Serum samples collected from LASV-infected NHPs at regular intervals after the infection were monitored for the presence of infectious virus using a standard 50% tissue culture infectious dose (TCID_50_) assay. (**B** and **C**) Data are represented as connecting lines for each individual animal. At the time of necropsy, fluids (*n* = 3) (**B**) and solid organs (*n* = 17) (**C**) were collected for quantification of infectious virus titers using a standard TCID_50_ assay. (**B** and **C**) Colored lines represent the medians of each group, whereas colored circles are individual values. Dotted lines represent the limit of detection of the assay. LN, lymph node.

All 10 NHPs had readily detectable infectious LASV in serum samples between days 4 and 7 after challenge. The extent of infectious virus in serum was indistinguishable across the groups, with peak titers of 1 × 10^6^ and 1 × 10^7^ TCID_50_/mL achieved at the terminal endpoints (Figure 7, A and B). Similarly, infectious assays conducted on 20 fluid and organ specimens collected at the time of necropsy revealed similar end point viral titers across the 2 depletion groups as well as the control animals (Figure 7, B and C). The detection of LASV RNA in fluids and tissues yielded similar results, highlighting the fact that T cell depletion did not affect viral burden (Supplemental Figure 6).

### Histopathology.

**Figure 8:**
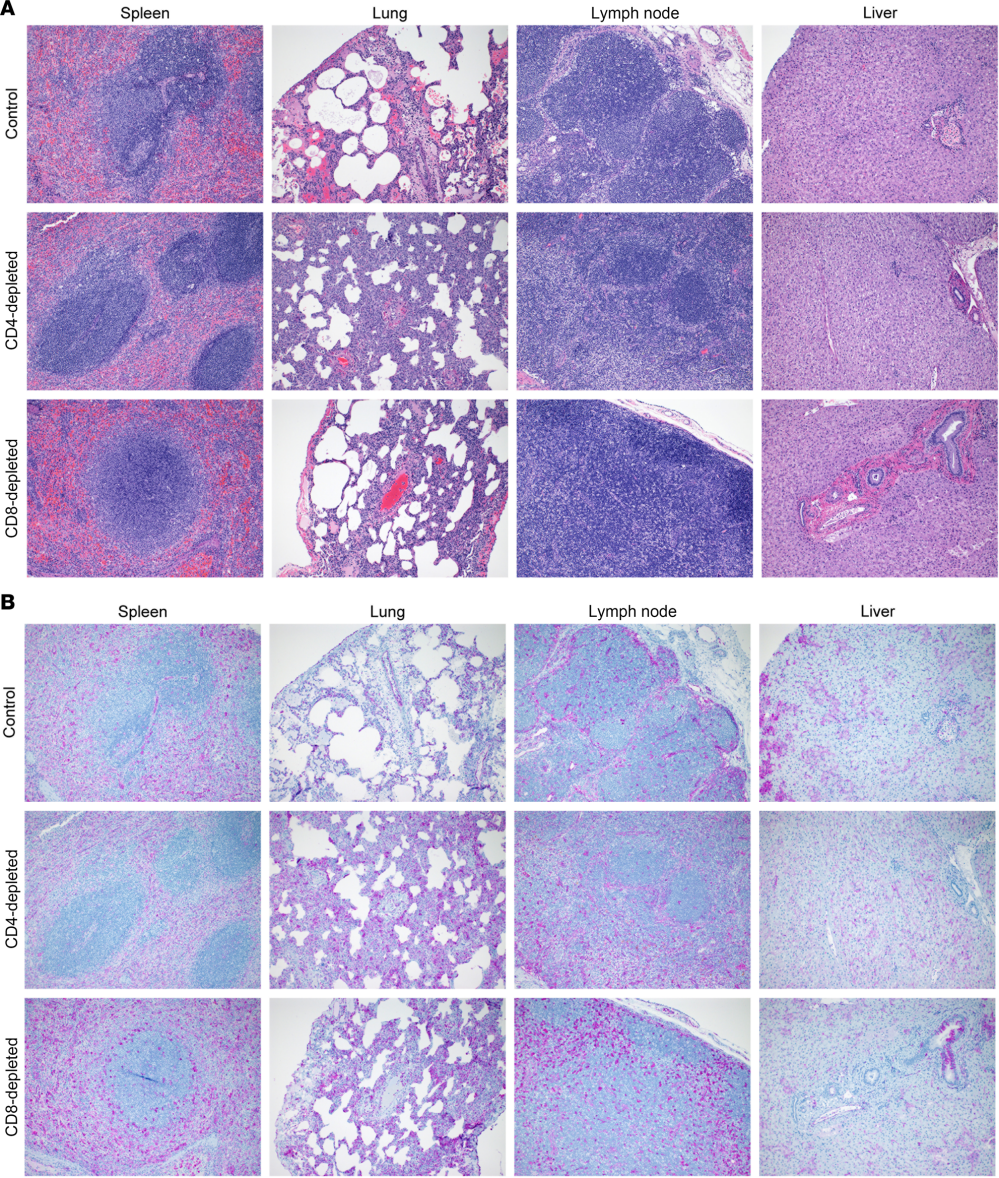
Histopathology of tissues from T cell–depleted LASV-infected cynomolgus macaques. (**A**) Formalin-fixed tissues (spleen, lung, lymph node, liver) from LASV-infected cynomolgus macaques were stained with H&E. (**B**) Formalin-fixed tissues (spleen, lung, lymph node, liver) from LASV-infected cynomolgus macaques were stained with rabbit anti-LASV nucleocapsid polyclonal antibody (purple) and counterstained with hematoxylin. Representative sections are shown. Total original magnification, ×100.

Histopathologic changes consistent with previously published models of LASV disease in NHPs were observed in all groups (21, 32) (Figure 8A). Lymphoid depletion in splenic follicles was most prominent (mild) in control NHPs with less prevalence in the CD8-depletion group (*n* = 2/3 mild, *n* = 1/3 minimal) and the least lymphoid depletion noted in the CD4 depletion group (*n* = 1/3 minimal). Pulmonary lesions, consisting primarily of interstitial pneumonia, alveolar edema, perivascular edema, and leukocyte infiltrates spilling into alveolar spaces, followed a similar pattern. Mild to moderate pulmonary lesions were observed in both evaluated NHPs in the control group, whereas moderate lesions were observed in 66% (*n* = 2/3) of the CD8 depletion group. CD4-depleted NHPs had the least severe pulmonary changes with minimal to mild pulmonary changes observed in 100% (*n* = 3/3) of the macaques. Interestingly, pulmonary lesions identically mimicked splenic follicular changes in that NHPs with greater lymphoid depletion had the most severe pulmonary lesions. This included both control animals as well as 2 NHPs examined from the CD8-depletion group.

Epididymitis was observed in a singular sampled epididymis from the CD8-depletion group. No significant histopathologic lesions were observed in evaluated sections of testis. Additional histopathologic changes in peripheral lymph nodes and liver are consistent with previously described NHP models of LASV infection and did not follow a discernable pattern in regard to depletion. Within lymph nodes, mild to moderate sinus histiocytosis with perinodal lymphohistiocytic inflammation, erythrophagocytosis, and lymphocytolysis were noted. Liver abnormalities consisted of minimal hepatocellular necrosis and sinusoidal fibrin thrombosis. Specific histopathologic changes in the urinary bladder, small intestine, large intestine, or pancreas were not observed beyond what would be considered background in these animals.

Viral antigen was detected in all major evaluated organs: spleen, lung, lymph node, and liver in control, CD4-depleted, and CD8-depleted NHPs (Figure 8B). Briefly, viral antigen was observed primarily in red pulp and parafollicular regions of the spleen, within bronchiolar epithelium, alveolar epithelium, endothelial cells, and alveolar macrophages of the lung; within macrophages of subcapsular, cortical, and medullary sinuses of the lymph node; and within biliary epithelium, endothelium, Kupffer cells, and lower numbers of hepatocytes of the liver.

## Discussion

The role of T cells in LF pathogenesis remains unclear, with several studies providing compelling evidence both supporting and opposing a role for immunopathogenesis. Mouse models offer the most compelling data toward at least a dual role for T cells, which includes involvement in immune-mediated pathogenesis of LF. Control of LASV replication in mice appears to be MHC dependent, with MHC-I–deficient mice (lacking CD8^+^ T cell responses) developing prolonged LASV infection, whereas MHC-II–deficient mice (lacking CD4^+^ T cell responses) are able to clear the infection (27). In an HHD mouse model, depletion of T cells alleviated lethal disease, but not LASV infection kinetics, possibly due to a dampened innate immune response (27). The specific role of CD8^+^ T cells in acute disease and LASV pathogenicity was further highlighted in 2 independent mouse models, chimeric IFNAR^–/–^ mice as well as STAT1^–/–^ mice, using antibody-mediated depletions. In the chimeric IFNAR^–/–^ model, mice depleted of CD8^+^ or CD8^+^ and CD4^+^ cells largely survived lethal LASV challenge, whereas those depleted for CD4^+^ cells succumbed to infection (28). Similar findings were observed in the STAT1^–/–^ mouse model where mice depleted for CD8^+^ or CD4^+^ and CD8^+^ T cells survived an otherwise lethal LASV challenge (29). Interestingly, similar studies in the STAT1^–/–^ model with ML29, a reassortant virus containing the small genomic segment of LASV, and the large genomic segment of Mopeia virus, a closely related but nonpathogenic virus, observed that both CD4^+^ and CD8^+^ T cells are responsible for pathogenicity (39). Nevertheless, the implications of CD8^+^ T cell involvement in LASV pathogenesis in mice aligns with similar work conducted with LCMV, the prototypic Old World Mammarenavirus (40).

In the current study, we sought to examine the role of T cells in the development of lethal LF disease in a NHP model that faithfully recapitulates severe human disease. Using an antibody-based depletion strategy, we successfully eliminated CD4^+^ or CD8^+^ T cells in groups of 4 NHPs (Figure 2). The effect of depletions extended beyond circulating T cells, with reductions also observed in lymphoid tissues by IHC analysis. The results of our study suggest that, individually, CD4^+^ or CD8^+^ T cells do not play a central role in disease severity of LASV infection. Additionally, we observed a depletion of circulating NK and NKT cells in the CD8-depleted group, which suggests that these other lymphocytes might also not be involved in the severity of LF in the NHP model (Figure 4 and Supplemental Figure 2). The absence of either cell type did not clinically alter the disease parameters observed in NHPs when compared with control, nondepleted animals, although slight delays in disease progression were noted in the CD4-depletion group and less lung pathology involvement were seen in both depleted groups (Figures 3 and 8, and Supplemental Figure 3). In alignment with the noted delay in lethal disease, clinical scores, including assessments of body temperature, for animals depleted for CD4^+^ T cells were slightly lower than those recorded for control or CD8-depleted animals (Figure 3). Nevertheless, infectious LASV titers from serial serum samples collected throughout the study were indistinguishable across the groups, as were most other parameters monitored, including hematological profiles, serum biochemistries, coagulation markers, and host responses (Figures 4–7). Furthermore, viral loads in tissues collected at the time of necropsy were all similar across the 3 groups, as assessed by infectious titration, quantitative PCR (qPCR), and anti-LASV IHC, and blinded histopathological analysis was unable to discern any observable differences in viral pathology (Figures 7 and 8, and Supplemental Figure 6). Combined, this multifaceted study does not support the findings of previous LASV disease modeling in murine models, and it suggests that T cells are not critical in the development of lethal LASV disease in the NHP model.

Survivors of LF generally have notable T cell responses upon resolution of disease, often with minimal neutralizing antibody responses, which supports a role for T cells in viral clearance (24–26). We noticed a similar phenotype in the NHP model, in which we observed a major increase of activated CD4^+^ and CD8^+^ T cells in the blood within the first week after infection (Figure 6, F and L), although levels of LASV-specific IgG antibody responses were low to undetectable in terminally ill animals (Figure 6R). Despite the high levels of T cell activation, it did not seem to affect the systemic viral replication and spread (Figure 7). This could be explained by the generation of an aberrant Th2/Tc2-biased response at 7 DPI, rather than the protective Th1/Tc1 response (Figure 6, I, J, O, and P). Indeed, type 1 T cell responses were previously shown to be predominant in LF survivors and strongly induced by protective LASV vaccines (25, 26, 35, 36, 38, 41–45). It is also interesting to note that previous studies on lethal LASV infection in NHP models have shown poor induction of Th1 responses from LASV-specific T cells (36, 38, 42, 46). A subsequent switch to Th1/Tc1 responses was observed at 10 DPI (Figure 6, I, J, O, and P), but this was probably too late since the infection was fatal at 11 DPI in control animals (Figure 3A). Another factor that could have contributed to the dysfunctional T cell responses is the abnormally elevated levels of activated T cells (average of 14% for CD4^+^ and 46% for CD8^+^), which could be indicative of bystander T cell activation, as previously suggested in humans and in mouse models (47). Overall, through the complete elimination of T cells prior to and after challenge during acute infection, our results suggest that neither LASV-specific nor bystander, nonspecific T cells are required for the development of lethal disease. However, it is worth noting that pulmonary lesions and leukocyte alveolar infiltration were more predominant in nondepleted control animals, suggesting a potential role of T cells in LASV-induced pathology (Figure 8A and Supplemental Figure 3). Although a double CD4 and CD8–depletion group was not included in the current study, the lack of significant difference between either the CD4- or CD8-depletion groups compared with the control nondepleted group imply the additional group was not warranted.

In addition to the lymphoid compartment, LASV infection appears to significantly affect the myeloid compartment. In response to the infection, we observed a large increase in CD16^+^ monocytes, typically more terminally differentiated activated populations of monocytes and known for their increased antigen presentation, antiviral activity, and their role in maintaining endothelial integrity (48). CD16^+^ monocytes were most likely elicited in response to the proinflammatory environment (Figure 6A and Supplemental Figure 5) and the endotheliopathy/tissue damages caused by LASV infection (Figure 8A) (49). Previous studies have reported a similar increase in activated monocytes in response to LASV infection in NHPs, especially in fatal outcome (37, 50). This widespread activation and differentiation of monocytes has been suggested to be T cell dependant in LASV-infected mice, contributing to a deleterious innate inflammatory responses (27). However, we show that this is not the case in the NHP model, where CD4^+^ or CD8^+^ T cell depletion did not affect the generation of CD16^+^ monocytes (Figure 6, B–D). Interestingly, following the early activation phase, we also observed a decrease in total monocyte counts in the blood at later time points (Figure 4G), which could be a result of active LASV replication in this permissive cell type (27, 51).

The data presented here offer a benchmark for future vaccine studies, specifically those focusing on elucidating correlates of protection associated with LASV vaccines. Previously, Marzi and colleagues demonstrated antibody responses are crucial for protection afforded by a VSV-based Ebola virus vaccine in NHPs using an antibody-mediated T cell–depletion strategy during the immunization phase (30). In light of the existing mouse modeling data suggesting a role of T cells in LASV pathogenesis, a similar approach for studying correlates of protection associated with LF vaccines may not have been advisable. However, the current study addresses those caveats and suggests a similar approach for LF vaccines in NHPs is achievable.

## Methods

### Sex as a biological variable.

Due to availability, only male animals were included in the current study. LASV is not known to have a sex-based variability in humans or in disease models.

### Biosafety.

All work with infectious LASV and potentially infectious materials derived from animals was conducted the NML BSL 4 laboratory. Sample inactivation and removal was performed according to approved standard operating protocols.

### Animals.

Ten male cynomolgus macaques (*Macaca fascicularis*, Worldwide Primates Inc.) weighing between 3.0 and 3.4 kg were randomly divided into experimental groups for CD4^+^ T cell (*n* = 4) and CD8^+^ T cell (*n* = 4) depletions as well as a nondepleted control (*n* = 2) group. Animals were group housed, 2 per cage, in a devoted room in the NML biosafety level (BSL) 4 facility according to approved standard operating procedures for high containment. The holding room’s environment was rigorously controlled by computerized systems and was kept at 24°C ± 2°C with 50% humidity and 20 air changes every hour. Animals were fed commercially produced monkey chow (25% protein, LabDiet 5048) twice daily and had unrestricted access to drinking water. Environmental enrichment included toys, treats, vegetables, and fruit. All experimental manipulations and clinical exams were performed on sedated animals (8–10 mg/kg ketamine delivered by intramuscular injection) with supplemental isoflurane (1%–3%, delivered with medical oxygen) as needed.

### T cell depletion.

Animals were depleted for CD4^+^ and CD8^+^ T cells using a previously established strategy (Figure 1) (30). CD4^+^ T cell depletions were accomplished using a simianized anti-CD4 antibody (clone CD4R1; NHP reagent resource) administered at 50 mg/kg/treatment subcutaneously 7 days prior to challenge (–7 DPI) as well as intravenously 4 days and 1 day prior to challenge (–4 DPI, –1 DPI), and again 4 DPI. CD8^+^ T cell depletions were accomplished using a simianized anti-CD8α antibody (clone MT807R1; NHP reagent resource) administered at 10 mg/kg/treatment subcutaneously 7 days prior to challenge (–7 DPI) as well as intravenously 4 days and one day prior to challenge (–4 DPI, –1 DPI), and again 4 DPI. Prior to intravenous administration of antibodies, animals were given diphenhydramine (Benadryl, 2–5 mg/kg, 0.2mL) via intramuscular injection to minimize the risk of hypersensitivity reactions.

### Flow cytometry.

WBC composition and T cell activity was monitored on exam days prior to depletion as well as before and after LASV challenge in 100 μL of fresh EDTA-treated whole blood collected according to a published protocol (52). Some of the markers were changed for in-depth characterization of the T cell phenotypes. The full list of antibodies is shown in Supplemental Table 1. The flow cytometry gating strategy depicted in Supplemental Figure 1 was used to monitor T cell depletion, gating was done in FlowJo v10.10. Absolute counts were calculated by equating the live CD45 positive population to the WBC count from the HM5 instrument (see below) and using the following calculation: number of CD4^+^ or CD8^+^ T cells/number of live CD45^+^ cells × WBC counts.

### Anti-CD4 and anti-CD8 antibody ELISA.

The detection of anti-CD4 and anti-CD8 depleting antibodies in NHP serum was performed using an indirect ELISA assay. Half area well, high binding flat bottom plates (Corning) were coated with recombinant CD4 or CD8α proteins (Sino Biological) at 30 ng per well and incubated overnight at 4°C. Plates were washed with phosphate-buffered saline + 0.1% Tween20 (PBST) and were then incubated with blocking buffer (PBST + 5% skim milk) at 37°C for 1 hour. Using blocking buffer as a diluent, 4-fold serial dilution of serum samples (starting at 1:100) were added to appropriate wells and incubated at 37°C for 1 hour. Two-fold serial dilution of anti-CD4 or anti-CD8 depleting antibodies used above (from 32 to 0.5 ng/mL) were used as a standard curve to determine depleting antibody concentrations. Plates were washed with PBST, followed by the addition of horseradish peroxidase (HRP) conjugated goat anti-human IgG (H+L) secondary antibodies (#5220-0330, KPL) at a working concentration of 0.5 μg/mL, and incubated for 1 hour at 37°C. After incubation, plates were washed with PBST and HRP activity was quantified by using the TMB substrate (Thermo Fisher Scientific) before reading the optical density at 650 nm (OD_650_).

### Challenge experiment.

The challenge stock of LASV strain Josiah (passage 3) was cultured in Vero cells (CCL-81, ATCC) and titered using standard median tissue culture infective dose (TCID_50_) methods. The authenticity of the viral stock was confirmed by deep sequencing, matching previously published sequences for LASV strain Josiah, and was confirmed to be mycoplasma free. Depleted and control NHPs were challenged with 1 × 10^4^ TCID_50_’s of LASV strain Josiah (clade IV) by intramuscular injection, a dose that was previously determined to be 100% lethal in naive animals (21). After challenge, animals were evaluated at least twice daily for signs of disease by trained personnel using an approved scoring sheet (fever, posture, respiration, feces/urine, food intake, recumbence, attitude and skin turgor) as previously reported (53) Complete exams, including assessment of body weights, temperature, and respiration rates as well as collection of blood samples for blood biochemistries, differential blood count and virus detection, were conducted on days –14, –7, –4, –1, 4, 7, and 10 after challenge or at the time of necropsy. Terminally ill animals were euthanized by exsanguination while anesthetized (as above) in accordance with the Canadian Council on Animal Care (CCAC) guidelines and the recommendations of the Weatherall report. Necropsies were performed with collection of clinical specimens from lung, liver, heart, spleen, kidney, small and large intestines, thymus, adrenals, pancreas, testis, epididymis, bladder, vitreous humor, and brain as well as inguinal, axillary, and mesenteric lymph nodes for histologic analysis and viral titrations. Where present, pleural fluid was also collected.

### Hematology, biochemistry and coagulation.

Hematology was conducted on EDTA venous blood with the HM5 hematology analyzer (Abaxis) and the following parameters were evaluated: RBC, hemoglobin (Hb), hematocrit (HCT), mean corpuscular volume (MCV), mean corpuscular hemoglobin (MCH), mean corpuscular hemoglobin concentration (MCHC), red cell distribution weight (RDW), platelets (PLT), mean platelet volume (MPV), WBC, and neutrophil, lymphocyte, monocyte, eosinophil, and basophil counts (absolute and percentage for each). Serum biochemistries, including albumin (ALB), alkaline phosphatase (ALP), alanine aminotransferase (ALT), amylase (AMY), TBIL, blood urea nitrogen (BUN), calcium (CA), phosphorus (PHOS), creatinine (CRE), glucose (GLU), sodium (NA), potassium (K), TP, and globulin (GLOB), were analyzed on a VS2 biochemistry analyzer (Abaxis) using serum samples. Fibrinogen levels, PT, and aPTT were measured on citrate plasma using a Satellite Max analyzer (Stago). D-dimers were measured using an ELISA-based assay (Thermo Fisher Scientific)

### Cytokines.

Serum samples used for cytokine Luminex assays were inactivated by gamma-radiation (5 Mrad, Cobalt-60 source). Expression levels of cytokines and chemokines were assessed in NHP serum samples by using the ProcartaPlex Non-Human Primate Cytokine & Chemokine Panel 30plex (Thermo Fisher) according to the manufacturer’s instructions. The following cytokines and chemokines were targeted for the study: GM-CSF, IFN-γ, IL-1β, IL-2, IL-4, IL-5, IL-6, IL-8 (CXCL8), IL-10, IL-12p70, IL-13, IL-17A (CTLA-8), IL-18, IL-23, TNF-α, soluble CD40L, G-CSF (CSF-3), IFN-α, IL-1RA, IL-7, IL-15, BLC (CXCL13), Eotaxin (CCL11), IP-10 (CXCL10), I-TAC (CXCL11), MCP-1 (CCL2), MIG (CXCL9), MIP-1α (CCL3), MIP-1β (CCL4), and SDF-1α (CXCL12). Serum samples were diluted 1:8 and test plates were run using a Luminex MAGPIX instrument (Luminex). The final concentration of each analyte was expressed in pg/mL using the mean fluorescence intensity (MFI) rate. An integrated cytokine score (CytoScore) from the linear combination of all 30 analytes was calculated for each sample as previously described (54).

### LASV serological assays.

Seroconversion was assessed in acute serum samples collected at days 4, 7, and 10 days after challenge and at the time of euthanasia. Samples were tested for the presence of IgM and IgG antibodies reactive to the LASV nucleoprotein (NP) and prefusion glycoprotein (pfGP) using a commercial ELISA assay (Zalgen Labs). Neutralizing antibody titers were determined in serum samples collected at 10 days post-infection using a replication competent recombinant vesicular stomatitis virus which expresses the LASV glycoproteins in place of its own as well as the green fluorescent protein (VSVΔG/LASVGPC/GFP) by standard microneutralization assay.

### Virus detection.

Total RNA was extracted from blood, vitreous humor, pleural effusion or solid organs using guanidium thiocyanate lysis buffers (Qiagen) on a KingFisher Apex (Thermo Fisher) automated extraction platform with the MagMAX Viral/Pathogen Nucleic Acid Isolation Kit (Thermo Fisher), according to the manufacturer’s instructions. Extracts were tested for the presence of LASV RNA using primers (LASV-FWD: ATGGCTTGTTTGTTGAAGTCRAA and LASV-REV: TGACCAGGTGGATGCTAATTGA) and probe (LASV-G-P: 5′-CATGTCACAAAATTCTTCATCGTGCTTCTCA-3′) targeting the LASV glycoprotein gene on a QuantStudio 5 Real-Time PCR System. Infectious virus titers from tissue homogenates and blood/serum samples were determined in triplicate with 10-fold serial dilutions on Vero cells using standard TCID_50_ methodologies and the Reed-Muench formula.

### Histopathology.

Immediately after collection, tissue specimens were placed in individual cassettes and submerged in 10% neutral buffered formalin. After a minimum of 14 days fixation time, tissues were processed with a Sakura VIP-6 Tissue Tek, on a 12-hr automated schedule, using a graded series of ethanol, xylene, and PureAffin. Embedded tissues were sectioned at 5 μm and dried overnight at 42°C prior to staining with H&E according to standard histopathological methods.

Specific anti-LASV immunoreactivity was detected using Rabbit anti-Lassa Nucleoprotein protein (Cusabio, catalog CSB-PA318401LA01LNP) at a 1:1000 dilution. The ImmPRESS VR horse anti-rabbit IgG polymer (Vector Laboratories, catalog MP-6401) was used as a secondary antibody. For negative controls, replicate sections from each block were deparaffinized and stained in parallel following an identical protocol, with the primary antibody replaced by rabbit IgG (Vector Laboratories, catalog I-1000-5) at a dilution of 1:2,500. The tissues were stained using the DISCOVERY Ultra automated stainer (Ventana Medical Systems) with a DISCOVERY purple kit (Roche Tissue Diagnostics, catalog 760-229).

CD8 immunoreactivity was detected using a rabbit polyclonal primary antibody to CD8/CD8α/Leu-2 (2GV6) (Sino Biological, catalog 10980-T24) at a 1:1000 dilution. The DISCOVERY Omnimap anti-rabbit HRP (Roche Tissue Diagnostics, catalog 760-4311) was used as a secondary antibody. CD8 was stained with a DISCOVERY purple kit (Roche Tissue Diagnostics, catalog 760-229). CD4 immunoreactivity was detected using a rabbit monoclonal primary antibody to CD4 (Abcam, catalog ab133616) at a 1:50 dilution. An anti-rabbit NP (Roche Tissue Diagnostics, catalog 760-4817) and anti-NP AP (Roche Tissue Diagnostics, catalog 760-4827) were used as the secondary detection system. CD4 was stained with a with a DISCOVERY Yellow kit (Roche Tissue Diagnostics, catalog 760-239). For negative controls for each antibody, replicate sections from each control block were stained in parallel following an identical protocol, with the primary antibody replaced by rabbit IgG (Vector Laboratories, catalog I-1000-5) at a dilution of 1:2,500.

### Statistics.

Survival rates were compared using Fischer’s Exact test in R v4.4.1. Time-to-death comparisons (global and pairwise) were done using the log-rank test corrected for multiple comparisons with the Holm-Sidak method in GraphPad Prism v10.5.0. For the T cell depletion, concentrations of 0 were changed to a value of 0.01 cells/μL (representing 1 cell in 100 μL of blood). Concentrations were log_10_-transformed and a linear model with a group × day interaction was used for each cell type. This analysis was done in R v4.4.1, data manipulation used the tidyverse package (v2.0.0).

### Study approval.

Animal experiments were approved by the IACUC of the Canadian Science Centre of Human and Animal Health and performed following the guidelines of the CCAC in an CCAC-approved facility.

### Data availability.

All data for this study can be found within the manuscript or in the accompanying supplemental files.

## Author contributions

JP, JA, KR, HF, and DS conceived and designed the study; JP, NT, GS, JA, YD, RV, JPS, KT, KA, DK, CSC, and DS executed the study as well as collected and analyzed data. JP and DS wrote the first draft of the manuscript, which was reviewed and edited by all authors.

## Funding support

This work is the result of NIH funding, in whole or in part, and is subject to the NIH Public Access Policy. Through acceptance of this federal funding, the NIH has been given a right to make the work publicly available in PubMed Central.

The Public Health Agency of Canada

CIHR-CEPI Leadership Award for Excellence in Vaccine Research for Infectious Diseases of Epidemic Potential (DS)

The Intramural Research Program of the NIH

## Supplementary Material

Supplemental data

Supporting data values

## Version 1 09/25/2025

In-Press Preview

## Version 2 11/24/2025

Electronic publication
