# Supplementary material for: CD4+ and CD8+ T cells are not the main driver of Lassa fever pathogenesis in macaques
Source: JCI Insight. 2025 Sep 25;10(22):e199235. doi: 10.1172/jci.insight.199235 (PMC12643512; doi:10.1172/jci.insight.199235)
Supplement: Supplemental data [file jciinsight-10-199235-s224.pdf]

Table S1. Antibody panel for flow cytometry assays.

Supplier	Antibody	Clone	Channel	Catalog #
BD Biosciences	CD45	D058-1283	BUV395	564099
BD Biosciences	CD3	SP34-2	BUV496	741183
BD Biosciences	CD8	RPA-T8	BUV563	612914
BD Biosciences	CD16	3G8	BUV737	612786
BD Biosciences	CD45RA	5H9	BV421	740083
BD Biosciences	CCR6	11A9	BV480	566130
BD Biosciences	CD4	L200	BV605	562843
BD Biosciences	CD14	M5E2	BV650	563419
BioLegend	CD39	A1	BV711	328228
BioLegend	CXCR3	G025H7	BV785	353738
BD Biosciences	CD25	M-A251	BB515	565096
Beckman Coulter	NKG2A	Z199	PE	IM3291U
BD Biosciences	CCR7	2-L1-A	PE-CF594	566768
BioLegend	HLA-DR	L243	PE/Fire640	307676
Miltenyi Biotec	CD66abce	TET2	PerCP-Vio700	130-119-850
BD Biosciences	CCR4	1G1	BB700	566475
BD Biosciences	CD69	FN50	PE-Cy7	557745
BD Biosciences	TCRγδ	B1	APC	555718
BD Biosciences	CD20	2H7	Alexa 700	560631

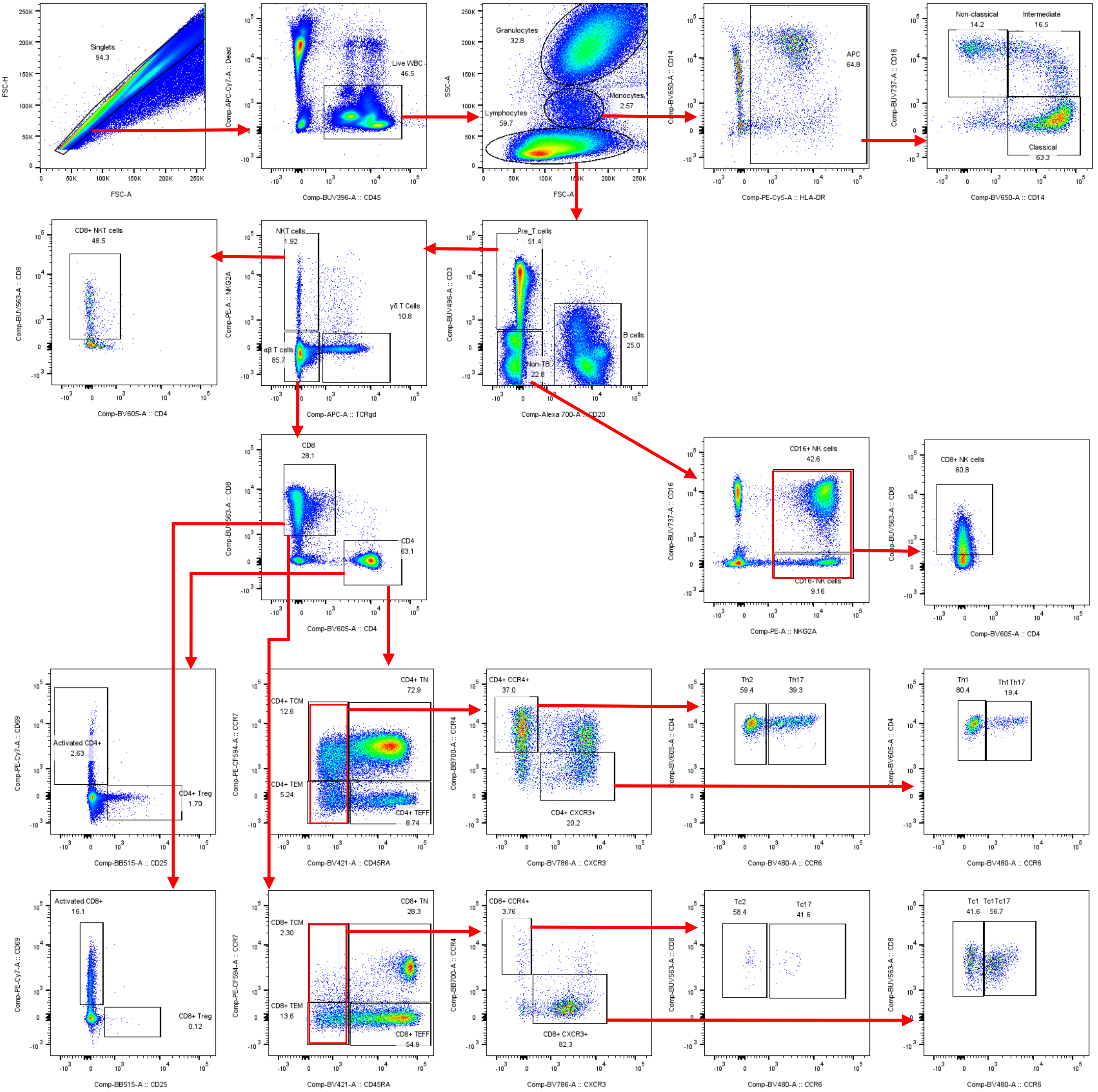

Figure S1. Flow cytometry gating strategy. Representative flow cytometry gating strategy with a 19-color panel (see table S1) using whole blood samples from cynomolgus macaques. CD4+ T cells, CD8+ T cells, B cells, NK cells, NKT cells, γδT cells and monocytes were gated using FlowJo 10.8.1 software following acquisition on a FACSymphony A5 cytometer. APC, antigen-presenting cells; Tc, cytotoxic T cells, TCM, central memory T cells; TEFF, effector T cells; TEM, effector memory T cells; Th, helper T cells, TN, naïve T cells; Treg, regulatory T cells; WBC, white blood cells.

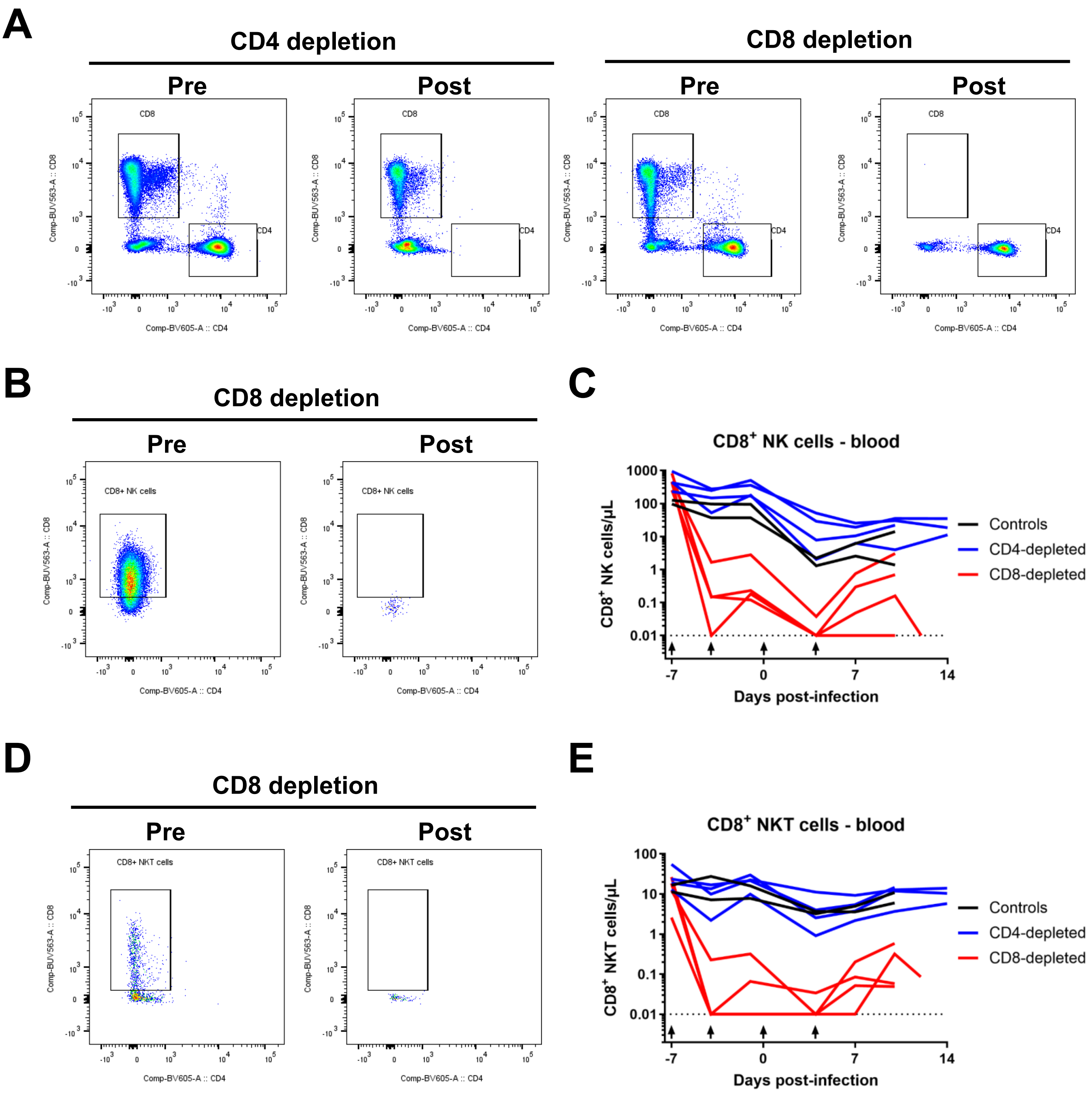

Figure S2. Antibody-mediated depletion of lymphocyte populations in cynomolgus macaques. (A) Representative flow cytometry plot showing the CD4+ and CD8+ T cells populations pre- (day -7) and post- (day -4) administration of CD4 or CD8-depleting antibodies. (B) Representative flow cytometry plot showing the CD8+ NK cells populations pre- (day -7) and post- (day -4) administration of CD8-depleting antibodies. (C) Absolute counts of circulating CD8+ NK cells were monitored from fresh EDTA-treated whole blood during routine exams (-7, -4, -1, 4, 7, and 10 days post-infection) and terminal necropsy exams using flow cytometry. (D) Representative flow cytometry plot showing the CD8+ NKT cells populations pre- (day -7) and post- (day -4) administration of CD8-depleting antibodies. (E) Absolute counts of circulating CD8+ NK cells were monitored from fresh EDTA-treated whole blood during routine exams (-7, -4, -1, 4, 7, and 10 days post-infection) and terminal necropsy exams using flow cytometry. (C, E) Arrows indicate the days when depleting antibodies were administered. Data are represented as connecting lines for each individual animal.

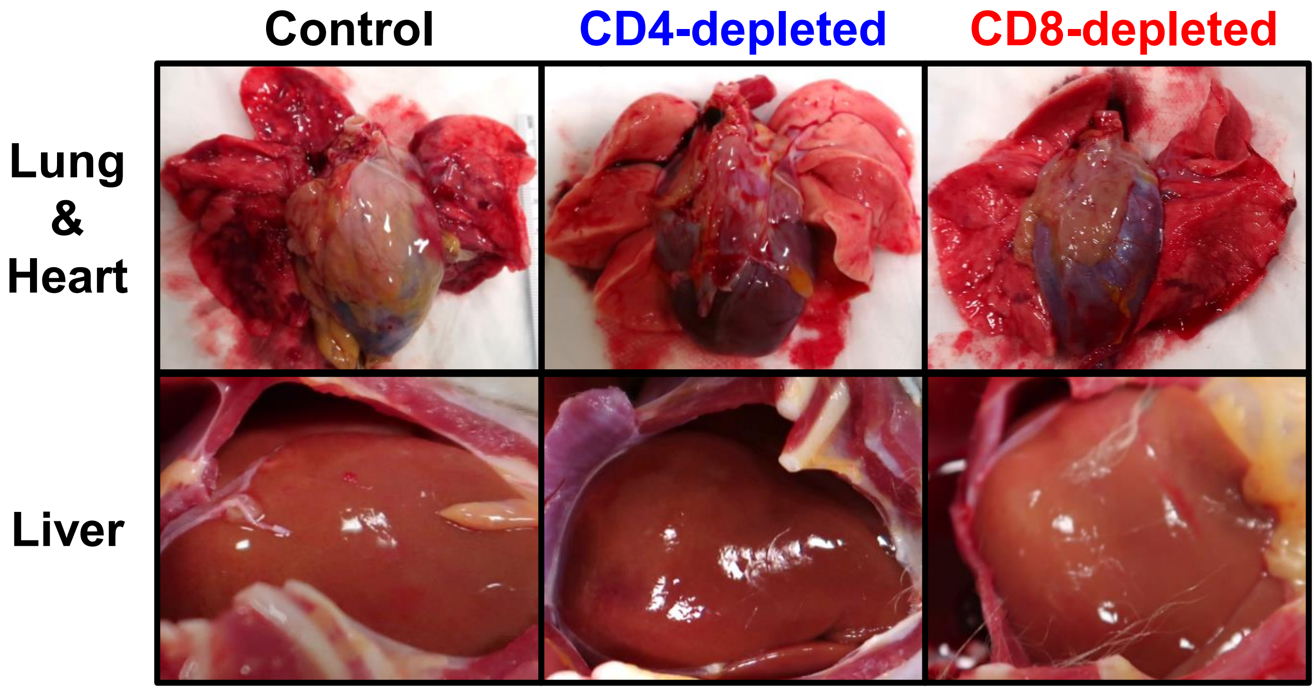

Figure S3. Gross pathology of T cell-depleted LASV-infected cynomolgus macaques. Macroscopic identification of pathological changes in lung, heart and liver tissues was performed post-mortem on terminally ill LASV-infected cynomolgus macaques. Representative images from CD4-depleted, CD8-depleted or control LASV-infected NHPs are depicted. Pulmonary hemorrhage and hepatic pallor were generally milder in T cell-depleted animals.

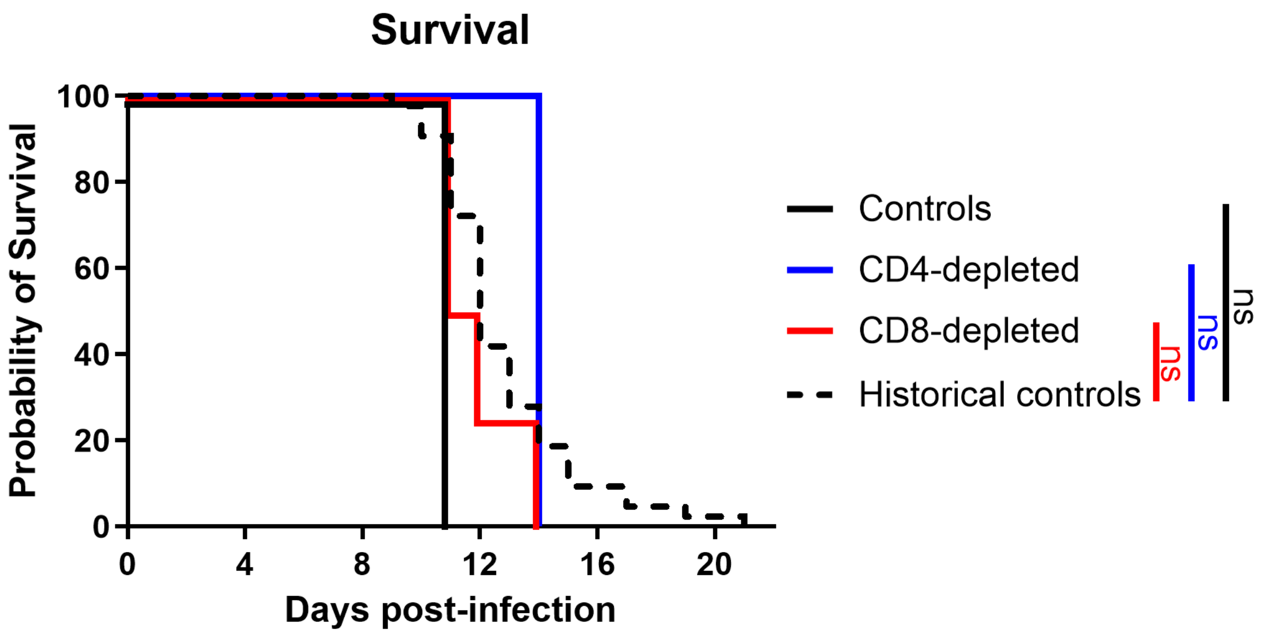

Figure S4. Survival analyses of T cell-depleted LASV-infected cynomolgus macaques compared to historical controls.

Kaplan-Meier survival curves from CD4-depleted, CD8-depleted and control groups of LASV-infected cynomolgus macaques were compared with historical controls (cynomolgus macaques infected with LASV Josiah [n=43]) from 10 previous studies (PMID: 15971954, 21548931, 23303805, 28869611, 29882740 , 31071008, 31578242, 33398113, 34634087, 36906645). Statistical significance was calculated a log-rank test with a Holm-Sidak post-test (ns, non-significant).

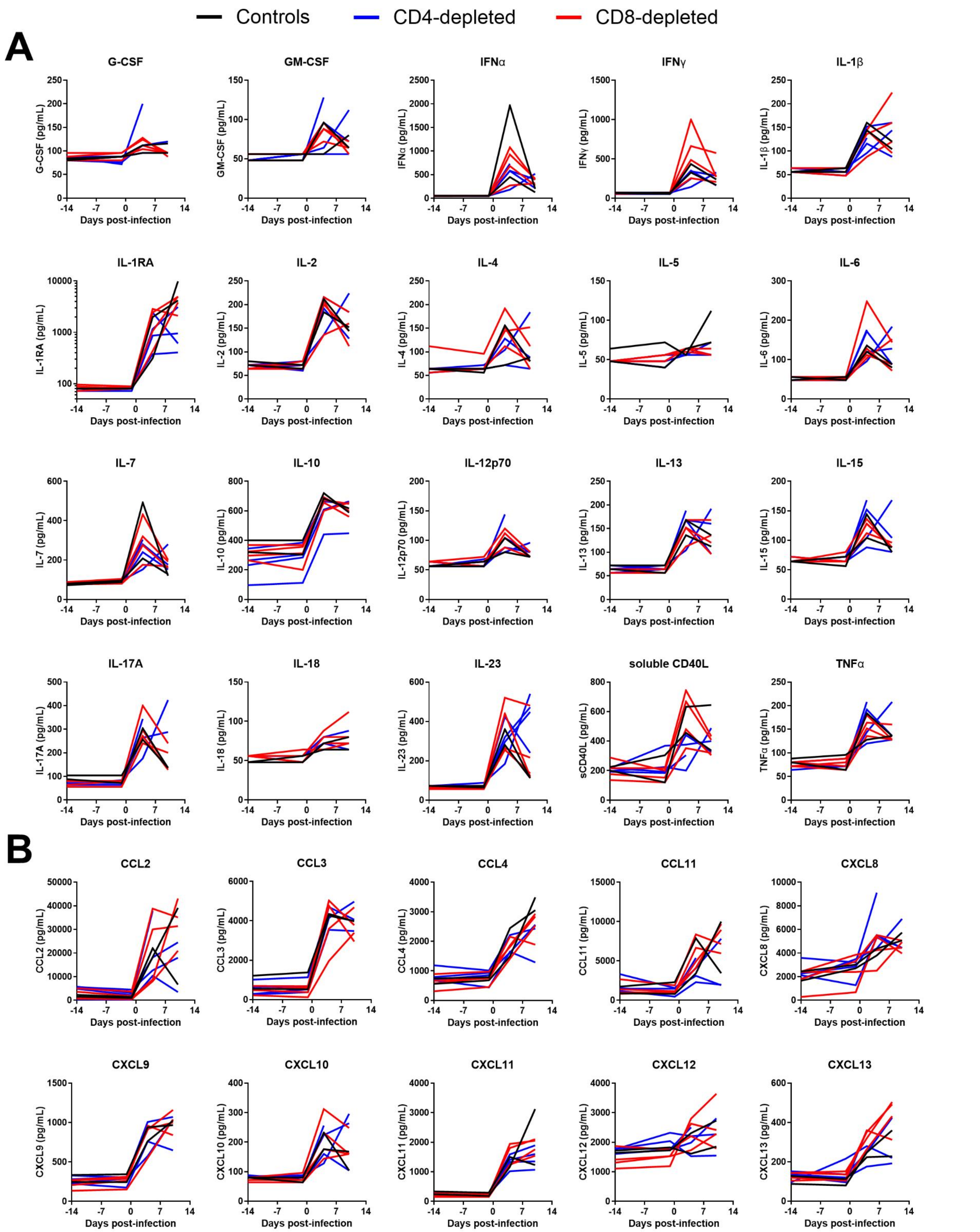

Figure S5. Cytokine and chemokine responses in T cell-depleted LASV-infected cynomolgus macaques. Serum samples collected from LASV-infected NHPs at regular intervals pre- and post-infection were monitored for the presence of (A) cytokines (G-CSF, GM-CSF, IFNα, IFNγ, IL-1β, IL-1RA, IL-2, IL-4, IL-5, IL-6, IL-7, IL-10, IL-12p70, IL-13, IL-15, IL-17A, IL-18, IL-23, soluble CD40L, TNFα), and (B) chemokines (CCL2, CCL3, CCL4,CCL11, CXCL8, CXCL9, CXCL10, CXCL11, CXCL12, CXCL13) using a multiplex fluorescent bead-based immunoassay. Data are represented as connecting lines for each individual animal.

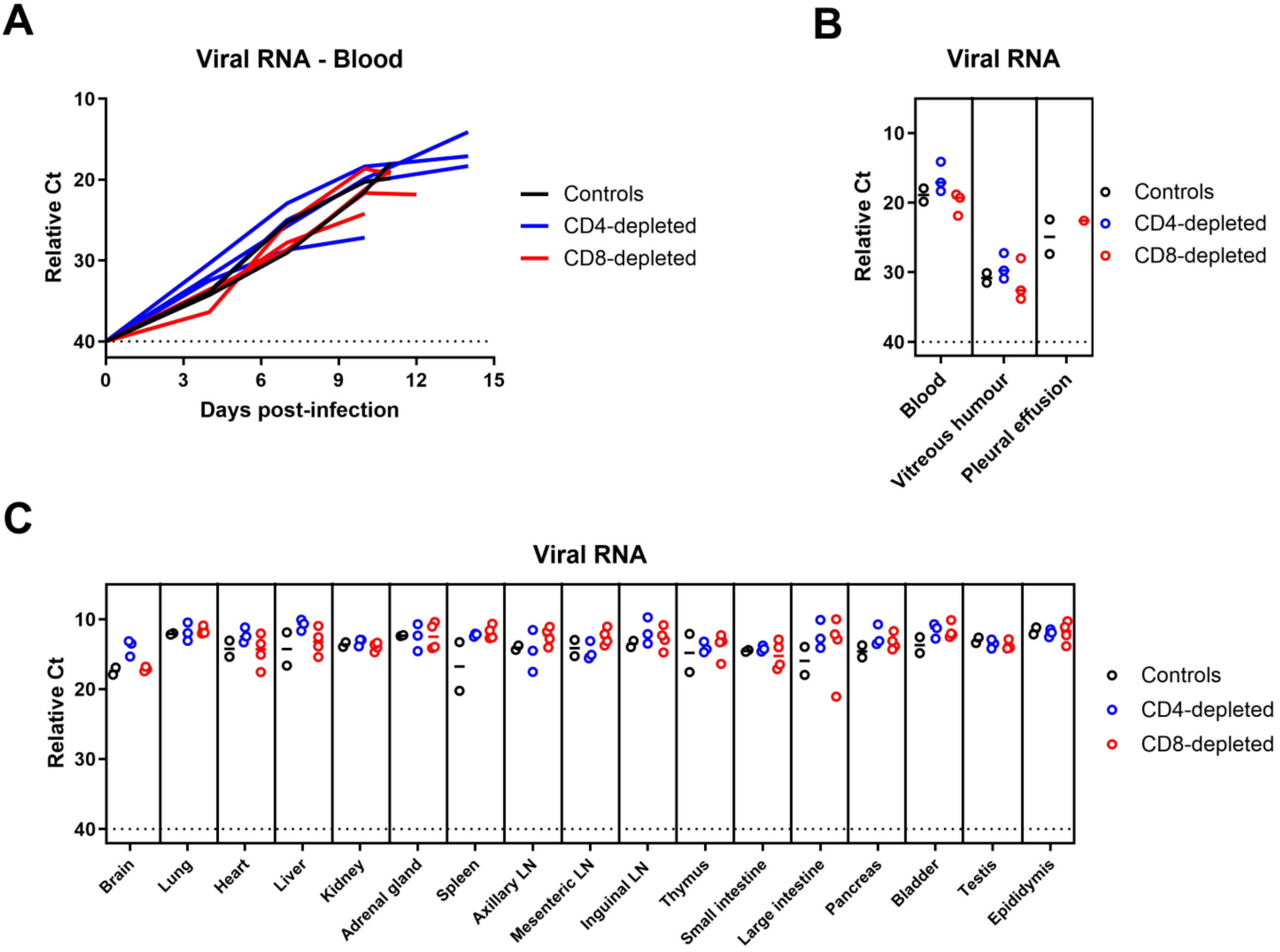

Figure S6. Viral RNAemia in T cell-depleted LASV-infected cynomolgus macaques. (A) EDTA-treated blood samples collected from LASV-infected NHPs at regular intervals after the infection were monitored for the presence of LASV RNA using a reverse-transcription quantitative polymerase chain reaction assay (RT-qPCR) assay. Data are represented as connecting lines for each individual animal. At the time of terminal necropsy, (B) fluids (n=3) and (C) solid organs (n=17) were collected for quantification of viral RNA using an RT-qPCR assay. (B-C) Colored lines represent the medians of each group, whereas colored circles are individual values. Dotted lines represent the limit of detection of the assay. LN, lymph node.
